# Comprehensive review of clinical presentation, treatment, and prognostic factors of airway burns

**DOI:** 10.25122/jml-2025-0081

**Published:** 2025-05

**Authors:** Rares-Adrian Giurgiu, Eliza-Maria Bordeanu-Diaconescu, Andreea Grosu-Bularda, Adrian Frunza, Sabina Grama, Cătălina-Ştefania Dumitru, Raducu-Andrei Costache, Carina-Ioana Cristescu, Ioan Lascar, Cristian-Sorin Hariga

**Affiliations:** 1 Department 11, Discipline Plastic and Reconstructive Surgery, University of Medicine and Pharmacy Carol Davila, Bucharest, Romania; 2 Burn Centre, Emergency Clinical Hospital of Bucharest, Bucharest, Romania

**Keywords:** inhalation injury, burns, smoke systemic poisoning, pulmonary dysfunction

## Abstract

Inhalation injury is a major contributor to poor outcomes in burn patients, increasing the risk of respiratory complications, prolonged hospitalization, and mortality. This review summarizes current knowledge on the pathophysiology, diagnosis, and management of airway burns, based on clinical studies and guidelines. Injuries may be supraglottic, subglottic, or systemic, each leading to inflammation, airway obstruction, and impaired gas exchange. Carbon monoxide and cyanide toxicity further worsen systemic hypoxia. Diagnosis depends on clinical signs, imaging, and bronchoscopy, which remains the gold standard. Treatment involves airway stabilization, ventilatory support, inhaled therapies, and antidotes for toxic exposure. Prognosis is affected by burn extent, systemic response, and comorbidities such as substance abuse. Survivors often experience long-term pulmonary dysfunction, emphasizing the need for early, multidisciplinary intervention.

## INTRODUCTION

Inhalation injuries are a severe complication of burns, often accompanying extensive skin damage and significantly increasing morbidity and mortality. These injuries raise the risk of pulmonary complications [1], and even isolated airway burns can lead to long-term lung dysfunction [2]. While some studies suggest they do not affect immediate survival [3], overall, long-term survival is lower in affected patients [4]. Mortality correlates with total body surface area burned and patient age, which also increases the likelihood of airway injury [4]. Recognizing their severity, European and American guidelines classify suspected inhalation injuries as a criterion for referral to specialized burn centers [5]. Incidence figures vary with setting and diagnostic criteria, ranging from ≈10 % in U.S. registries [6], through 0.3–43 % in European studies [7], to 4.9–11.3 % in recent Chinese reports [8].

## THE PATHOPHYSIOLOGY OF INHALATION TRAUMA

The pathophysiology and evolution of airway burns differ according to the injury site and the agent causing the injury. Thus, anatomically, inhalation injuries are divided into supraglottic, subglottic, and systemic poisoning. The most important aetiologic agents of airway injuries are heat, carbon monoxide poisoning, and cyanide.

### Supraglottic burns

Although temperatures can reach up to 500°C in an enclosed space fire, the low heat capacity of air, efficient heat dissipation in the upper airway, and reflex glottic closure usually limit true thermal injury to the airway above the carina [9]. Burns at this level can cause massive edema of the tongue, epiglottis, and aryepiglottic folds, which will cause airway obstruction. This edema may develop rapidly during fluid resuscitation, so the respiratory status should be continuously monitored, and initial assessment is not sufficient [1]. Preventive intubation should be considered if history and clinical examination raise the suspicion of airway burns.

### Subglottic burns

Subglottic burns are typically caused by inhaled chemical toxins from burning materials [9,10], as superheated air alone, except for steam, does not reach this level. The composition of smoke influences the severity of the resulting inflammation. Key pathophysiologic changes include mucosal hyperemia, bronchospasm, fibrin and mucus plug formation, desquamation, surfactant loss, and impaired mucociliary clearance [9]. These lead to alveolar collapse [11], reduced oxygenation, and increased susceptibility to infection [10]. Toxins trigger bronchial inflammation, edema, and obstruction by debris and secretions, causing ventilation-perfusion mismatch, systemic hypoxia, and heightened infection risk [10].

When the airways are obstructed in a particular region, the tidal volume redistributes to the unobstructed areas, which injures these regions through barotrauma and volumetric trauma, leading to acute respiratory distress syndrome (ARDS) and pneumothorax. All these changes affect the ventilation/oxygenation rate and result in right-left shunting [12]. ARDS represents the most severe lower-airway injury, resulting in noncardiogenic pulmonary oedema, CO_2_ retention, and marked hypoxaemia, often followed by multi-organ failure and death [13]. In a conscious, mechanically unventilated patient, tachypnoea and dyspnoea will be severe. According to the Berlin definition, ARDS is acute (onset within 7 days of a precipitating event), not fully explained by heart failure or fluid overload, requires positive end-expiratory pressure (PEEP) ≥ 5 cmH_2_O, and is classified by the ratio of arterial oxygen partial pressure to fraction of inspired oxygen (PaO_2_/FiO_2_): mild (200–300 mmHg), moderate (100–200 mmHg) and severe (< 100 mmHg) [14].

### Systemic poisoning

Inhalation injuries cause systemic effects like hypoxemia, hypercapnia, acidosis, and widespread inflammation due to lung-derived mediators [1]. Additionally, toxic combustion products like carbon monoxide (CO) and cyanide contribute to systemic damage. CO, a colorless and odorless gas from incomplete combustion, is a leading cause of death at the scene in burn victims [1].

CO binds to haemoglobin (Hb) with approximately 200-fold greater affinity than oxygen (O_2_), causing the hemoglobin dissociation curve to shift to the left [9]. O_2_ supply to tissues is low due to compromised transport capacity and tissue-independent dissociation [10]. CO competitively inhibits the intracellular cytochrome enzyme system, particularly cytochrome P-450, resulting in the inability of cellular systems to utilize O_2_.

Hydrocyanic acid (hydrogen cyanide) also inhibits the cytochrome oxidase system and has a synergistic effect with carbon monoxide, producing tissue hypoxia, acidosis, and decreased oxygenation to the brain [1].

The biological half-life of carboxyhaemoglobin (COHb) is roughly 250 minutes when breathing room air, decreases to 40–60 minutes with 100 % O_2_, and can be further shortened by hyperbaric oxygen therapy. However, logistical challenges of monitoring and fluid resuscitation within a hyperbaric chamber often limit its use [15].

## DIAGNOSIS OF INHALATION INJURY

Despite the particularly important role that inhalation injury plays in the outcome and survival of the burned patient, to date, there is no consensus on the diagnosis, staging, and prognosis of airway burns [16]. The anamnesis should be oriented to the identification of the burned material, the duration of exposure, the time elapsed since the burn occurred and how the smoke exposure was realized (confined or open fire/explosion, domestic or industrial accident), the time elapsed until the casualty was rescued or removed from the confined space [16,17].

Immediately after trauma, the signs and symptoms of airway burns may be minimal or non-existent. The progression, however, may be very rapid toward airway obstruction, which is life-threatening [18]. Suspicious signs of airway injury include facial burns, hoarse voice, burned vibrissae, sputum, and sooty oral cavity [17]. Facial edema, stridor, wheezing, dyspnea, and cyanosis are signs of advanced respiratory injury [4]. However, clinical examination alone can be misleading, and correlation with other investigations is necessary. One study showed that although 70% of patients with airway burns had facial burns, 70% of patients with facial burns did not also have significant lung injury [10].

Upon arrival at the emergency department, patients should undergo a standardized assessment including complete blood count, ionogram, arterial blood gas, creatinine, pulse oximetry, ECG, and heart-lung X-ray. Ideally, tests for carboxyhaemoglobin and cyanide in the blood will be performed [19]. In most cases, the initial radiograph and blood gas will be normal on admission or at most slightly altered, but they will undergo significant changes in evolution [4]. Computed tomography (CT) can detect distal airway injuries not visible on bronchoscopy; peribronchial ground-glass opacities may appear within three hours of inhalation trauma [20]. Supraglottic burns are diagnosed by direct visualization on clinical exam, and flexible laryngoscopy can assess vocal-cord injury when bronchoscopy is not immediately available [21]. Other possible imaging investigations that may confirm the diagnosis of airway burns would be technetium-99 or xenon computed tomography, but these investigations are very rare and expensive and are therefore not routinely used to diagnose airway injury [9].

However, history and clinical examination are subjective diagnostic tools compared to bronchoscopy [18]. Used in the diagnosis of airway burns since 1975 [22], flexible bronchoscopy (fibrobronchoscopy) is not yet widely available, but is widely used for rapid and reliable diagnosis of inhalational injuries [10]. Flexible bronchoscopy allows direct visualization of both the supraglottic floor and subglottic structures. Fibrobronchoscopy can identify and extract soot and other foreign bodies from the airways. It is also a useful tool for performing bronchoalveolar lavage, obtaining samples for bacteriologic culture, and initiating targeted antimicrobial therapy [23]. Flexible bronchoscopy is recommended for confirming cases of inhalational injuries in both high and low clinical suspicion patients, being an investigation with a low complication rate [24].

The severity of inhalational injury can be categorized, according to the Abbreviated Injury Score (AIS) scale (Table 1), which is usually correlated with: increased mortality [25] in some studies, but not always [26], a decrease in oxygenation [27], but also with inconsistent results [26] and an increase in the duration of mechanical ventilation [26]. However, not all studies could validate this conclusion [24].

**Table 1:** Abbreviated Injury Score (AIS) system for fibrobronchoscopic staging of inhalation injury

Grade	Class	Description
0	No lesions	Absence of sooty deposits, erythema, edema, bronchorrhea, or obstruction
1	Mild lesions	Small or patchy areas of erythema, sooty deposits in proximal or distal bronchi
2	Moderate lesions	Moderate erythema, sooty deposits, bronchorrhea, or bronchial obstruction
3	Severe lesions	Severe inflammation with friable mucosa, significant soot deposits, bronchorrhea, or obstruction
4	Massive injury	Mucosal desquamation, necrosis, and complete obliteration of the lumen

Virtual bronchoscopy using computed tomography does not offer the benefits of fibrobronchoscopy and has not been established in current practice [24].

## TREATMENT

As with all burns, therapeutic maneuvers should be started at the site of the trauma. The victim must be evacuated and decontaminated immediately. All clothing, as well as rings, watches, and jewelry, should be removed immediately, as they may be contaminated with toxins (hydrogen cyanide, for example), retain heat, or have a constrictive, tourniquet effect as edema develops [17]. A rapid primary survey following Advanced Life Support (ALS) principles (Airway, Breathing, Circulation) should identify and address any immediately life-threatening issues [16]. Securing the airway by intubation may be difficult at the trauma scene, depending on the skills of the first aid team and the existing airway edema [16]. It has been observed that the majority of intubations of burn patients in the United States (almost 75%) are performed in a pre-hospital setting by staff inexperienced in burn patient management [28]. For patients without obvious ventilatory disturbances at the trauma site, it may be prudent to delay intubation until arrival in the emergency department, where the necessary expertise and access to all the necessary tools for proper airway management are available. Given the possibility of multiple complications of intubating patients, the risk of intubation may outweigh the benefit [28]. In the case of victims who are short of breath, the decision to intubate becomes much more difficult. If transportation time to the hospital is short, mask ventilation with a humidified O_2_ reservoir may be a temporary solution. Obtaining and securing at least one peripheral venous pathway is mandatory, and fluid resuscitation should be started at the trauma site [17]. Subsequently, a second, more thorough assessment should be performed, and ideally, information should be obtained regarding medical history, allergies, and other concomitant injuries [16,17]. A general clinical examination, including a neurological examination [16,17], should also be performed.

### Upper airway management

The risk of airway obstruction with loss of ventilation and oxygenation of the patient increases with time, as airway patency may decrease even with minimal airway burns, the individual response to the insult being highly variable [29]. Airway edema develops progressively, usually within the first 72 h, and is the result of a combination of local trauma, cutaneous burns, and fluid resuscitation [29]. Ideally, any stable patient with suspected airway burn should receive laryngoscopy, chest radiography, arterial blood gas analysis, and blood carbon monoxide determination in the emergency department in addition to the standard clinical examination [30]. If any of these investigations are pathologic, the airway should be secured by emergency orotracheal intubation [30]. Fibrobronchoscopy for evaluation of the lower airway should be performed only after securing the airway by intubation, ventilation control, surgical dressing of skin burns, and initial management of other coexisting injuries [30].

In the case of airway injury, airway edema will increase with fluid resuscitation; therefore, it is vital to secure the airway and control ventilation, even if the patient is conscious and not in respiratory failure at presentation. Although all patients with suspected airway burns are considered potential difficult intubation patients [30], if orotracheal intubation is delayed, it will become even more difficult because of severe upper airway edema that will develop after the start of fluid resuscitation [31].

Tracheostomy gives a long-lasting airway approach with increased patient comfort. A survey of 129 burn units facilities in the United States and Canada found that tracheostomy was performed, on average, two weeks after admission [32], despite an existing consensus for performing tracheostomy earlier than 2 weeks, with the general recommendation being in favor of tracheostomy for all patients requiring intubation longer than 7 days [33].

### Lower airway management

Therapeutic measures considered for inhalation injuries in subglottic airways should ameliorate the main pathophysiologic changes of this condition: mucosal edema and hyperemia, expectoration of mucus and airway deposits, bronchospasm, and improvement of the ventilation/perfusion ratio. These measures must also not further injure the respiratory system through complications such as barotrauma [10].

Multiple experimental studies have shown that nebulization with agents that reduce the blood flow in the bronchial artery may be effective by reducing edema [34]. In theory, limiting fluid administration might also lessen pulmonary swelling, but clinical data show that under-resuscitation increases mortality, while excessive fluids raise the risk of ARDS in patients with large total surface-area burns [35]. Although the Parkland formula remains widely used (including in our centre), many units now employ computer-based algorithms to individualize fluid goals [36]. However, fluid administration should be titrated according to the diuresis value to avoid under- and overloading patients with fluids.

While traditionally used to diagnose lung infections, bronchoalveolar lavage is also effective for clearing pseudomembranes and airway debris, especially when performed repeatedly. Inhalation injury patients with pneumonia who underwent at least one fibrobronchoscopy had lower mortality, shorter ICU stays, and reduced hospitalization costs [37].

Airway clearance through the breakdown and expectoration of mucus and debris is central to treating subglottic burns. Due to the procoagulant effect of smoke inhalation, nebulized anticoagulants like heparin are beneficial [38]—they degrade fibrin deposits [39], prevent microvascular clotting [1], and have been shown to reduce mortality [1,9]. N-acetylcysteine, a mucolytic and antioxidant, promotes mucus clearance and is typically alternated with heparin every 4 hours [9]. Because it may trigger bronchospasm, it is administered with bronchodilators such as albuterol. This combined protocol has improved oxygenation, reduced ventilation days, and enhanced survival without increasing pneumonia or bleeding risk [40,41]. β2-agonists also help by relaxing airway smooth muscle, reducing inflammation, and supporting mucociliary function [42]. Epinephrine further improves oxygenation by reducing airway edema and bronchial constriction [43,44].

Bronchial hygiene is essential for clearing secretions, debris, and pathogens from the airways. It includes therapeutic coughing, deep breathing every 2 hours, chest physiotherapy (e.g., percussion, vibration), suctioning, and early mobilization [17]. Turning the patient every 4 hours [17] and using adjuncts like bronchoscopy, mucolytics, suctioning of secretions, and certain ventilation modes can further aid clearance [45].

Mechanical ventilation in burn patients is challenging due to hypercapnia, reduced chest compliance, and airway injury. It is indicated in cases of respiratory distress, hypoxemia (PaO_2_ <65 mmHg), hypercapnia (PaCO_2_ >50 mmHg), or a PaO_2_/FiO_2_ ratio <200 [46]. Inhalation injuries increase airway resistance and reduce lung compliance, raising the risk of barotrauma and ventilator-induced lung injury, which can progress to ARDS. Ventilation strategies should maintain oxygenation and airway patency while minimizing additional lung damage [46].

### Management of systemic poisoning

In confined space fires with large smoke releases, not only do physical burns of the airways pose difficulties of care, but also poisoning from inhaled smoke inhalants such as carbon monoxide and cyanides. Treatment for these poisonings must be quick, as these substances have harmful systemic effects that can be fatal.

CO poisoning warrants emergency intervention when COHb levels exceed 10%. The amount of carboxyhaemoglobin in the blood can be measured by arterial blood gas measurement, thus quantifying the carbon monoxide level. The biological half-life of COHb depends on the inspired oxygen fraction: approximately 320 minutes on room air and 74 minutes with 100 % O_2_ [19]. Hyperbaric oxygen therapy (HBOT) can further shorten the COHb half-life by up to 20 minutes [19], although systematic reviews have not demonstrated clear superiority of HBOT over high-flow normobaric oxygen [47]. First-line treatment consists of 100 % O_2_ delivered via a high-flow non-rebreather mask or, if already intubated, through the endotracheal tube [48]. However, in patients in whom carboxyhaemoglobin levels remain elevated after oxygen administration, or whose mental status deteriorates, hyperbaric oxygen therapy is recommended, if the facility is available [49].

Smoke from combustion often also contains cyanides, especially from the combustion of plastics. Hydroxycobalamin, a natural derivative of vitamin B12, is the antidote of choice in Europe for the treatment of cyanide poisoning [36]. Intravenous administration of 5g of hydroxycobalamin over 15 min to patients with inhalational injuries has been correlated with a decrease in the number of pneumonias, a reduction in the number of days on mechanical ventilation, and a decrease in the duration that patients required intensive care [50]. Having minimal adverse effects, it can be administered empirically to all patients with suspected airway burns [50]. Another antidote used in cyanide poisoning is thiosulfate, but this treatment is less effective than hydroxycobalamin [17].

### Antimicrobial therapy

Respiratory tract infections are the most common complications in individuals with burn injuries. Pneumonia development is common in burn patients, especially after 72 hours of hospitalization. Major risk factors include inhalation injury and endotracheal intubation [21,37].

Pneumonia associated with mechanical ventilation is a common complication of patients with inhalational injuries [51]. The initial antibiotic therapy is often empiric and later adjusted according to the antibiogram of positive cultures from bronchial aspirate or bronchoalveolar lavage fluid.

A decrease in the incidence of pneumonia was not observed in patients who received prophylactic antibiotic therapy, but one study observed a decrease in mortality among these patients [52]. However, prophylactic antibiotic therapy is associated with an increase in the number of antibiotic multidrug-resistant bacteria [53], which ultimately leads to increased mortality. Therefore, the recommended therapeutic strategy is obtaining serial cultures from bronchial secretions or bronchoalveolar lavage fluid for surveillance and initiating early targeted antimicrobial therapy in associated pneumonia [53].

Knowledge about airway microbiota after inhalation injury and its impact on prognosis is limited [54]. One study found that patients with early hypoxia showed increased levels of facultative anaerobes, such as *Streptococcaceae, Enterobacteriaceae*, and *Staphylococcaceae*, compared to those without hypoxia [55]. Additionally, mechanically ventilated patients show a progressive decline in lung microbiota diversity, which is linked to respiratory infections [56].

## PROGNOSIS AND RECOVERY OF PATIENTS WITH INHALATION INJURY

Inhalation injury worsens burn outcomes, increasing overall mortality by up to 20 % compared to skin burns alone [57]. It raises the risk of respiratory failure, ARDS, and pneumonia. When pneumonia occurs alongside inhalation injury, mortality can reach 60% [9]. Even with a limited burn surface, airway burns significantly increase the risk of death [58]. Recent data have identified several predictors of poor outcome in inhalation-injured patients: larger total-burned surface area, development of ARDS, elevated lactate levels, leukocytosis, and derangements in liver and kidney function tests [59]. Severe inhalation injury was associated with decreased red blood cell counts, hemoglobin, platelets, and albumin. Leukocytosis >20.91×10_9_/L indicated severity, lactic acid >9.6 mmol/L predicted ARDS, and hemoglobin <8.3 mg/dL predicted mortality [59]. Other studies have similarly linked elevated inflammatory markers, neutrophil infiltration [60], and low PaO_2_/FiO_2_ ratios [61] with increased mortality. Substance abuse, especially alcohol, also exacerbates risk: even patients with < 5 % total-body-surface-area burns who test positive for drugs or alcohol face higher rates of intubation, respiratory failure, longer intensive-care stays, and extended hospitalizations [62]. Recovery from airway burns is prolonged, and long-term complications are common. Survivors may have reduced lung capacity even decades later [63]. Pulmonary rehabilitation can aid recovery [64]. Dysphagia is also significantly more frequent, up to 16 times, in patients with airway burns compared to those with only skin burns [65]. A study of 830 burn patients found that inhalation injuries increased in-hospital mortality but had no impact on post-discharge mortality or readmission rates [66]. Another study reported similar long-term health outcomes, though return-to-work rates at 24 months were lower in those with inhalation injuries [67].

## CONCLUSION

Inhalation injuries significantly worsen the prognosis of burn patients, increasing the risk of respiratory failure, systemic toxicity, and long-term complications. Early recognition, prompt airway management, and targeted therapies are essential to reduce mortality and improve outcomes. Despite advances in supportive care, the diagnosis and treatment of inhalation injuries remain challenging, highlighting the need for standardized protocols and continued research to optimize patient recovery.
